# THP-1-derived polarized macrophages differ by drug transporter transcriptomics, P-glycoprotein activity and rifampicin uptake kinetics

**DOI:** 10.1007/s00204-025-04270-2

**Published:** 2026-01-09

**Authors:** Katharina Hamburg, Cindy Bay, Jürgen Burhenne, Johanna Weiss, Julia C. Stingl, Dirk Theile

**Affiliations:** Internal Medicine IX - Department of Clinical Pharmacology and Pharmacoepidemiology Medical Faculty Heidelberg, Heidelberg University Hospital, Im Neuenheimer Feld 410, 69120 Heidelberg, Germany

**Keywords:** Tuberculosis, THP-1, Macrophages, Polarization, Drug transporters, P-glycoprotein, Rifampicin

## Abstract

Inferior efficacy of antibiotics (e.g. rifampicin) in M2 macrophage-dominant tuberculosis granuloma might be related to poor drug uptake into these cells, potentially mediated by altered expression and activity of drug transporters such as P-glycoprotein (P-gp, encoded by *ABCB1*). Consequently, THP-1 cells were differentiated (200 nM phorbol 12-myristate 13-acetate; 72 h) and polarized to M1 (50 ng/mL lipopolysaccharide, 20 ng/mL interferon-gamma; 48 h) or M2 (20 ng/mL interleukin-4, interleukin-13; 48 h) macrophages. Then, quantitative polymerase-chain reaction array-based transcriptional analysis, flow cytometry, and ultra-performance liquid chromatography coupled to tandem mass spectrometry were used to evaluate the impact of differentiation and polarization on the expression levels of 84 drug transporter genes, the efflux activity of P-gp, and the cellular rifampicin uptake kinetics. *ABCB1* was enhanced 166-fold during M1 polarization and 55-fold during M2 polarization. P-gp efflux activity in M2 cells was 1.55-fold higher than in M1 cells (*P* < 0.05). Rifampicin uptake into M2 cells was lower than into M1 cells after 2 h (*P* < 0.01) or 4 h exposure (*P* = 0.06) to 0.05 µM rifampicin. Together, when compared to M1 macrophages, M2 cells exhibit a considerably altered fingerprint of drug transporter expression levels, including an enhanced expression and activity of P-gp, being accompanied by lowered rifampicin uptake.

**Supplementary Information:**

The online version contains supplementary material available at 10.1007/s00204-025-04270-2.

## Introduction

Pro-inflammatory M1 and anti-inflammatory M2 macrophages play major roles in our immune defense against *Mycobacterium tuberculosis* (Mtb), the pathogen that causes tuberculosis. During Mtb infection, both macrophage phenotypes form a so-called granuloma together with other immune cells such as T cells to suppress further bacterial replication (Adams 1976; Bold & Ernst, 2009).

Previous work demonstrated that in patients with drug resistant tuberculosis granuloma, the M2 subtype is significantly more prominent than the M1 subtype (Cho et al. 2020), impeding successful bacterial eradication (Schreiber et al. 2009). Accordingly, the inferior antibiotics’ efficacy in M2-dominant granuloma might be related to poor drug uptake into M2 cells, potentially mediated by a variable expression and activity of drug transporters that are located in the cell’s membrane and modulate influx and efflux of various compounds. In fact, previous studies had already investigated if M1 and M2 cells differ by the expression of some selected drug transporters. For instance, the expression of ATP-driven drug transporters like breast cancer resistance protein (BCRP, coded by *ABCG2)* and multidrug resistance-related protein 1 (MRP1, coded by *ABCC1)* have already been investigated in M1 and M2 macrophages derived from the U937 monocyte cell line (He et al. 2018). Additionally, previous data indicate that U937 cells-derived M2 macrophages exhibit higher protein levels of the major drug transporter P-glycoprotein (P-gp, encoded by ABCB1), resulting in impaired lopinavir uptake into M2 cells compared to M1 macrophages (Cory et al. 2016). However, the net cellular drug uptake depends on the overall expression fingerprint of the whole transportome, comprising not only efflux (e.g. BCRP, MRP1, P-gp) but also uptake transporters (e.g. organic anion transporting polypeptide 1B1 (OATP1B1), encoded by solute carrier organic anion transporter family member 1B1 (SLCO1B1). In consequence, the work presented here is the first study to investigate the differences of THP-1 cells-derived M1 and M2 macrophages in terms of 84 different drug transporters. Additionally, special emphasis was taken on P-gp (an important transporter of rifampicin (Hartkoorn et al. 2007) and whether its efflux activity actually increases after polarization of monocytes to M2 macrophages. Furthermore, the uptake kinetics of rifampicin was compared in M1 and M2 macrophages.

Together, this works provides new data on drug transporter mRNA expression, P-gp activity, and rifampicin uptake into M1 and M2 macrophages.

## Materials and methods

### Materials

THP-1 cells were obtained from the American Type Culture Collection (ATCC, Manassas, USA) and RPMI 1640 Medium was purchased from PanBiotech (Aidenbach, Germany). Penicillin-streptomycin, fetal calf serum (FCS), phorbol 12-myristate 13-acetate (PMA), interferon gamma (IFNγ), lipopolysaccharides (LPS) from *E. coli*, interleukin 4 and 13, rifampicin, rifabutin, rhodamine 123, and zosuquidar (LY335979) were purchased from Sigma-Aldrich (Taufkirchen, Germany). RNeasy RNA isolation kit, Quantifast SYBR Green Master mix, RT^2^ First Strand Kit, RT^2^ SYBR Green qPCR Mastermix and RT^2^ profiler PCR Array (96-well format) human drug transporters were from Qiagen (Heiden, Germany). The RevertAiD h Minus First Strand cDNA Synthesis Kit, RIPA buffer, and the Micro BCA™ Protein-Assay-Kit were bought from Thermo Fisher (Dreieich, Germany). [^2^H_8_]-Rifampicin was obtained from AppliChem (Darmstadt, Germany). Protease inhibitors pefabloc, leupeptin, pepstatin and aprotinin were purchased from Carl Roth (Karlsruhe, Germany). ABSOLUTE™ QPCR SYBR^®^ Green Mix was from ABgene (Epsom, United Kingdom). Ammonia (28% in water), acetonitrile (ACN), formic acid (HCOOH), and methanol (MeOH) were purchased from Merck (Darmstadt, Germany). Ultra-purified water was provided from the arium^®^ mini ultrapure water system (Sartorius, Göttingen, Germany).

### THP-1 cells

Cells were cultured in RPMI 1640 medium supplemented with 10% FCS and penicillin (100 U/mLmL)-streptomycin (0.1 mg/mL) at 5% CO_2_ and 37 °C.

### Differentiation and polarization to M1/M2 macrophages

The differentiation protocol was chosen based on a previously published review (Mohd Yasin et al. 2022), including the 5-day resting phase following the differentiation using PMA (Daigneault et al. 2010; Chanput et al. 2014). Cytokine concentrations for polarization are based on the protocol from Shiratori and co-workers (Shiratori et al. 2017). According to these approaches, THP-1 monocytes were differentiated into M0 macrophages using 200 nM PMA for 72 h. After these 3 days of incubation, medium was changed. After resting for 5 days in drug-free medium, M0 macrophages were polarized for 48 h either into the pro-inflammatory M1 subtype using 50 ng/mL LPS and 20 ng/mL IFNγ, or the anti-inflammatory M2 subtype using 20 ng/mL interleukin 4 and 13 each.

### Validation of macrophage polarization

Polarization was validated by confirming the altered mRNA expression of tumor-necrosis-factor α (TNFα) and CC-chemokine ligand 22 (CCL22). mRNA of 10 × 10^6^ cells per biological replicate was isolated using the RNAeasy Kit. cDNA was synthesized with the RevertAid™ H Minus First Strand cDNA Synthesis Kit and quantitative real-time reverse transcription PCR (qPCR) was performed with the Quantifast SYBR Green Master Mix (Qiagen; for TNFα) or the ABSOLUTE™ QPCR SYBR^®^ Green Mix (ABgene) using a LightCycler^®^ 480 (Roche Applied Science, Mannheim, Germany) as described previously (Albermann et al. 2005).

PCR amplification was performed in 20 µL reaction volume containing 1:10 diluted cDNA, 1 x Absolute/Quantifast SYBR Green Mix and primers Primer sequences are displayed in supplementary table S1. Under the selected experimental conditions, glucuronidase β (GU) and ribosomal protein L13 (RPL 13) were the most suitable housekeeping genes for normalization in THP-1 monocytes, M1 and M2 cells, identified using geNorm (version 3.4, Center for Medical Genetics, Ghent, Belgium) (Vandesompele et al. 2002). Data was evaluated via calibrator-normalized relative quantification with efficiency correction using the LightCycler^®^ 480 software version 1.5.1.62 (Roche Applied Science, Mannheim, Germany). mRNA expression levels in M1 and M2 were normalized to THP-1 monocytes, which served as a control.

### Transcriptional changes of drug transporters during differentiation and polarization

To evaluate mRNA drug transporter expression in monocytes, differentiated M0 macrophages, and M1 and M2 cells, the RT^2^ Profiler™ PCR Array from Qiagen© with primers for 84 human drug transporters (see appendix) and reference gene primers was used. 1.5 µg of RNA was used for cDNA synthesis, using the RT^2^ First Strand Kit. For final PCR components mix, RT^2^ SYBR Green qPCR Mastermix solution, cDNA and RNase-free water were mixed according to manufacturer’s instructions. *Beta-actin* (*ACTB*), *beta-2-microglobulin* (*B2M*), *glycerinaldehyde-3-phosphate dehydrogenase* (*GAPDH*), *hypoxanthine phosphoribosyltransferase 1* (*HPRT1*), and *ribosomal protein lateral stalk subunit P0* (*RPLP0*) were chosen as reference genes and were also supplied by the instructor. The Roche LightCycler^®^ 480 (Roche Applied Science, Mannheim, Germany) was used for quantifying RNA expression by real-time RT-PCR and instructor’s conditions were applied. Again, data was evaluated via calibrator normalized relative quantification with efficiency correction using the LightCycler^®^ 480 software version 1.5.1.62 (Roche Applied Science, Mannheim, Germany) and final analysis was performed using the Qiagen online tool GeneGlobe. mRNA expression of drug transporters was normalized to reference genes and subsequently normalized to THP-1 monocytes, which again served as controls.

### Quantification of P-gp efflux activity after differentiation and polarization

For evaluation of P-gp efflux activity in THP-1 monocytes, differentiated M0 macrophages, and M1 and M2 cells, the rhodamine 123 efflux assay was used as described previously with minor modifications (Theile et al. 2013; Weiss et al. 2008; Nilles et al. 2023). Briefly, respective cells were incubated with 0.3 µM of the fluorescent P-gp substrate rhodamine 123 for 30 min at 37 °C under continuous shaking (rhodamine loading). Afterwards, cells were washed with 4 °C cold PBS and then either incubated with 1 µM of the specific P-gp inhibitor zosuquidar or left untreated for another 30 min at 37 °C under continuous shaking (rhodamine efflux phase). Subsequently, cells were washed two times and median fluorescence was recorded using a flow cytometer (MACSQuant analyzer 10, Miltenyi Biotec, Bergisch Gladbach, Germany). A total of 10,000 cells were used for analysis. The fluorescence ratio of zosuquidar-inhibited cells to non-inhibited cells of each phenotype was calculated and normalized to the respective ratio in THP-1 monocytes.

### Quantification of rifampicin uptake into polarized macrophages using ultra-performance liquid chromatography coupled to tandem mass spectrometry

For the analysis of cellular rifampicin uptake into M1 and M2 macrophages, our previously established UPLC-MS/MS methodology was adapted (Nilles et al. 2023). Alterations comprised the duration of chromatography (4 min vs. 4.5 min) to avoid carry over effects of rifampicin by increased rinsing. Cell lysis components were changed from methanol/water (50/50, v/v) to acetonitrile/water (50/50, v/v) to be able to use less volume of acetonitrile in the following step of protein precipitation. Therefore, the sample preparation protocol could be simplified from Eppendorf tubes to a 96-well plate format. Those changes in protocol were validated with regard to ICH M10 guideline to evaluate intracellular rifampicin concentrations in macrophages instead of the previously evaluated colon cancer cell line (LS180 cells). Validation was performed with 6 replicates of four different quality control samples (0.1 ng/mL;0.3 ng/mL, 37.5 ng/mL and 75 ng/mL) whose measured rifampicin concentration did not differ more than 15% (or 20% at the lower limit of quantification) from the calibration curve. The measurement system consisted of a triple stage quadrupole mass spectrometer (Waters Xevo TQ-S, Milford, MA, USA) and an Acquity Classic UPLC^®^ (Waters). Mass spectrometric analysis was performed by selective reaction monitoring using positive electrospray ionization.

For chromatography, the Waters Peptide BEH C18 Column (300 Å, 1.7 μm, 2.1 mm × 50 mm) at 60 °C was used. Mobile phase consisted of water/acetonitrile (95/5, v/v) + 0.1% formic acid (FA) (eluent A) and acetonitrile + 0.1% FA (eluent B). The separation gradient started with initial conditions of 80% A/20% B and changed after 3 min to 50% A/50% B. The eluent composition was adjusted after 3.1 min to 5% A/95% B and kept for 0.8 min to clean the system before returning to initial conditions for 0.4 min. The total run time was 4.5 min. Flow rate was set to a rate of 0.5 mL/min. and the total injection volume of each sample was 20 µL. The measurement was calibrated for a concentration range from 0.1 ng/mL up to 100 ng/mL.

### Sample preparation for UPLC-MS/MS analysis

M1 and M2 macrophages were differentiated and polarized in the same 96-well plates and treated with 0.05, 0.1, or 0.5 µM rifampicin for 1 h, 2 h, 4 h, or 6 h. After the end of the exposure time, cells were washed once with ice-cold PBS and stored at −20 °C until sample preparation for the UPLC-MS/MS measurement. Cell lysis of the samples was performed by using acetonitrile/water (50/50, v/v) with 5% ammonium hydroxide. Samples for calibration and quality control were treated with 10 µL of their corresponding working solution with known rifampicin concentrations. Uptake samples were treated with 10 µL of methanol/water (50/50, v/v) for volume compensation.

Then, the working solution of the internal standard was added to all samples with a final concentration of 600 ng/mL ^2^H_8_-rifampicin. For protein precipitation, acetonitrile was used. Afterwards, the plate was centrifuged for 10 min at 1,000 g and supernatant was transferred into a 96-well plate. There, the supernatant was evaporated with N_2_ (at 40 °C for 15 min) and resuspended with water/acetonitrile (50/50, v/v) + 0.1% FA.

## Normalization of intracellular rifampicin concentrations to total protein concentration

Protein samples of each cell type for each concentration and time point were harvested using lysis buffer, consisting of RIPA buffer and protease inhibitors. Determination of protein concentration for each sample was performed using the Micro BCA™ Protein-Assay-Kit according to manufacturer’s instructions. Intracellular rifampicin concentrations [ng/mL] were normalized to protein concentration [ng/mL] and resulted in [ng_Rifampicin_/ng_Protein_].

### Statistical analysis

The statistical analysis was performed using the GraphPad Prism 9.00 software (GraphPad Software, San Diego, USA). qPCR results of polarization markers were evaluated with Welch`s test. The effect of differentiation and polarization on drug transporter transcriptomics was evaluated by parametric, two-sided t-test. For statistics regarding P-gp function ANOVA with Tukey post-hoc test was performed. Rifampicin uptake differences between M1 and M2 macrophages was evaluated by Mann-Whitney test. A P-value < 0.05 was considered significant for all experiments except drug transporter transcriptomics, in which only a P value < 0.01 was considered significant.

## Results

### Macrophage differentiation and polarization

**Fig. 1:**
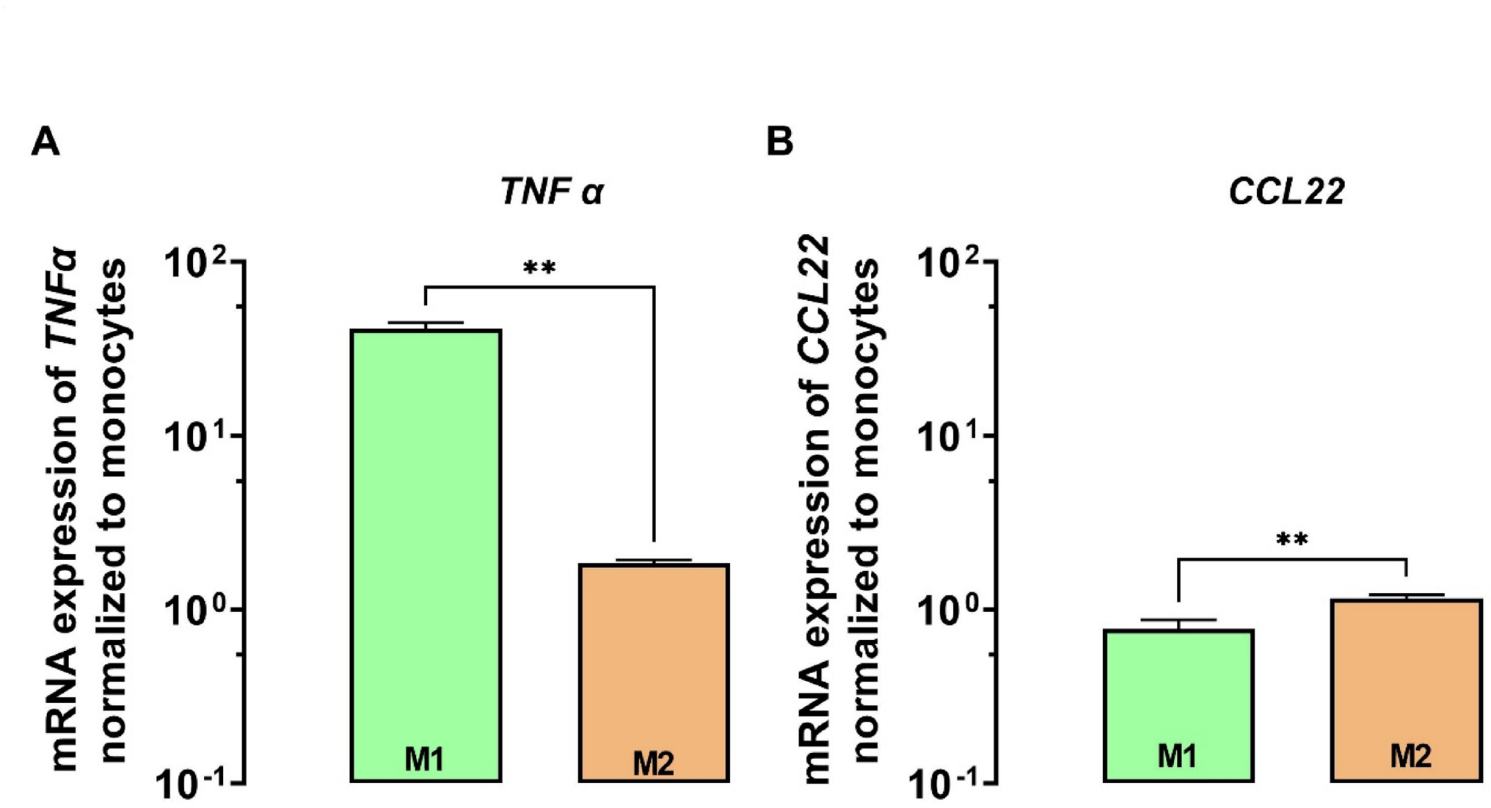
mRNA expression of specific M1 and M2 markers. *TNFα* (**A**) and *CCL22* (**B**) expression levels in M1 (green) and M2 (orange) macrophages, normalized to THP-1 monocytes. Data are shown as mean ± S.D. of biological triplicates. Statistical significance was determined by Welch’s test

 At first, we determined whether the correct macrophage phenotypes with our differentiation and polarization protocol was achieved. We therefore analyzed the expression of specific mRNA markers for the corresponding cell type and compared it to THP-1 cells. mRNA expression analysis demonstrated that the pro-inflammatory cytokine *TNFα* is 42-fold increased (*P* < 0,01; *n* = 3) in M1 macrophages, but only 1.85-fold in M2 cells. In contrast, mRNA of the anti-inflammatory *CCL22* is higher expressed in M2 macrophages (1.2-fold) than in M1 cells (0.8-fold) (Fig. 1).

### Drug transporter mRNA expression levels in differentiated and polarized macrophages

**Fig. 2:**
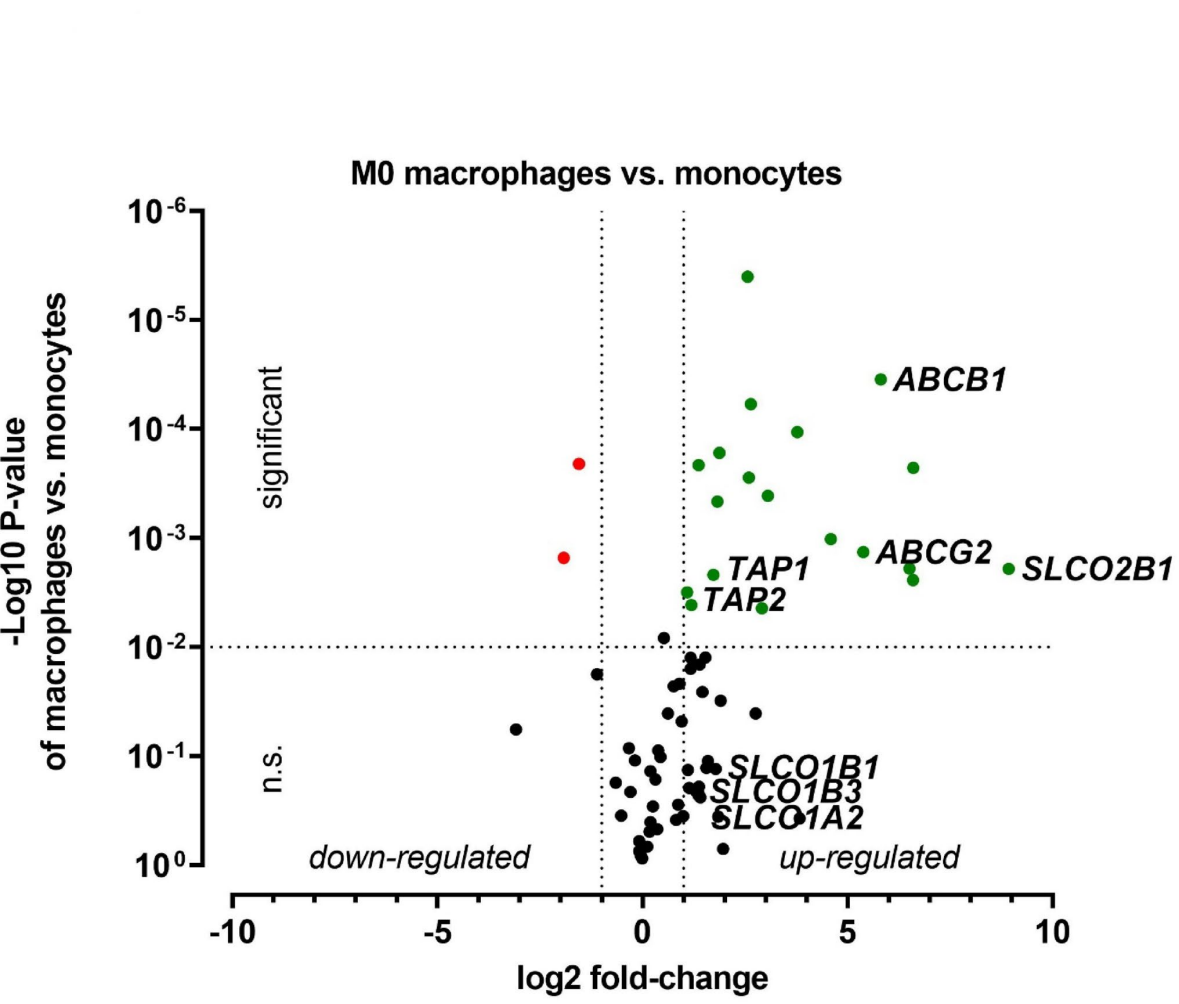
Drug transporter transcriptomics during differentiation from monocytes to M0 macrophages. Expression levels were normalized to monocytes. Drug transporters were marked as green dots, if their expression was significantly (P < 0.01) increased at least two-fold compared to the control and marked red when expression was significantly decreased at least two-fold. Gene names of selected drug transporters are highlighted. Data are shown as mean± S.D. of biological triplicates. Statistical significance was determined by parametric, two-sided t-test

**Fig. 3:**
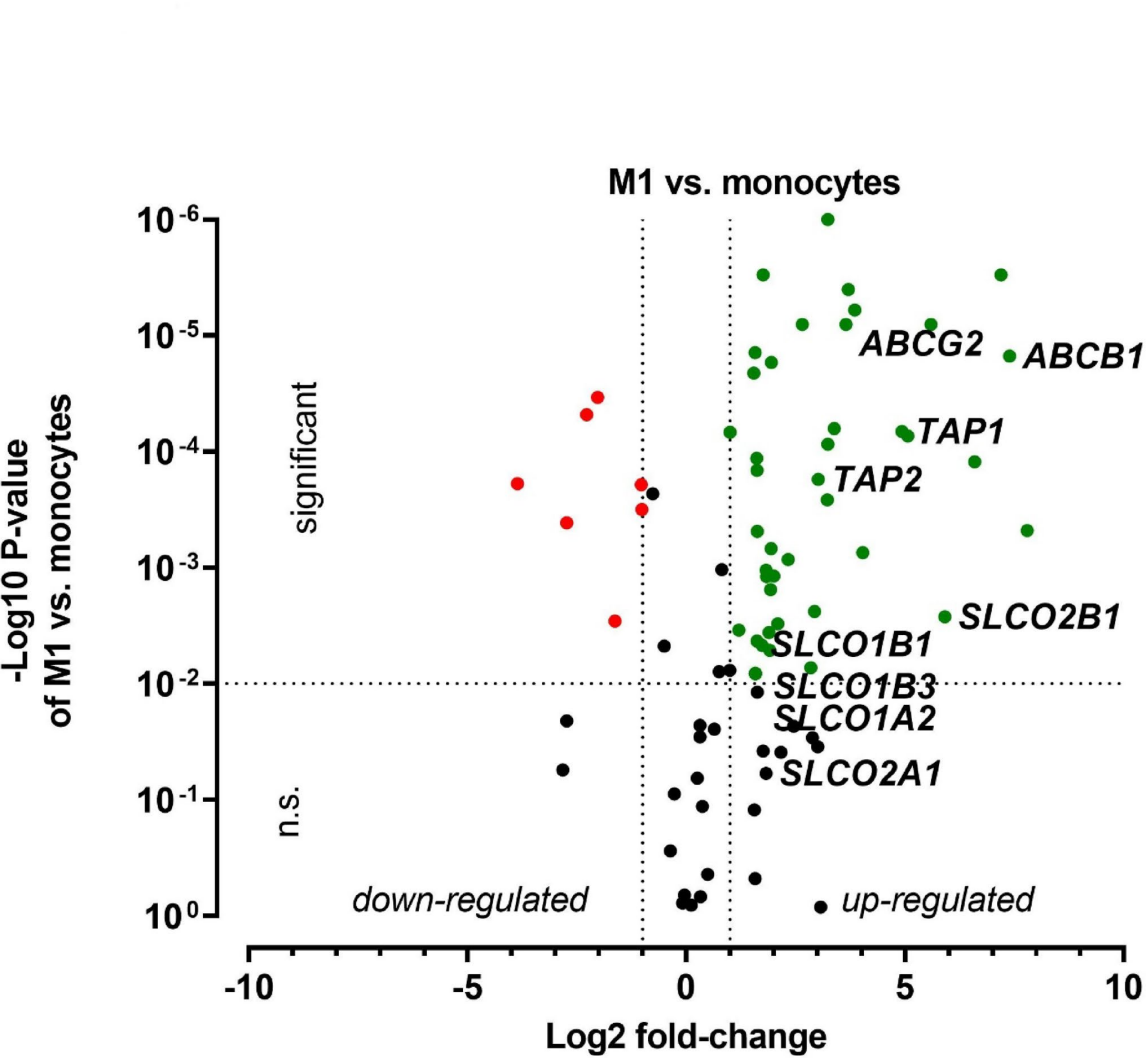
Drug transporter transcriptomics during polarization from monocytes to M1 macrophages. Expression levels were normalized to monocytes. Drug transporters were marked as green dots, if their expression was significantly (P < 0.01) increased at least two-fold compared to the control and marked red when expression was significantly decreased at least two-fold. Gene names of select drug transporters are highlighted. Data are shown as mean ± S.D. of biological triplicates. Statistical significance was determined by parametric, two-sided t-test

**Fig. 4:**
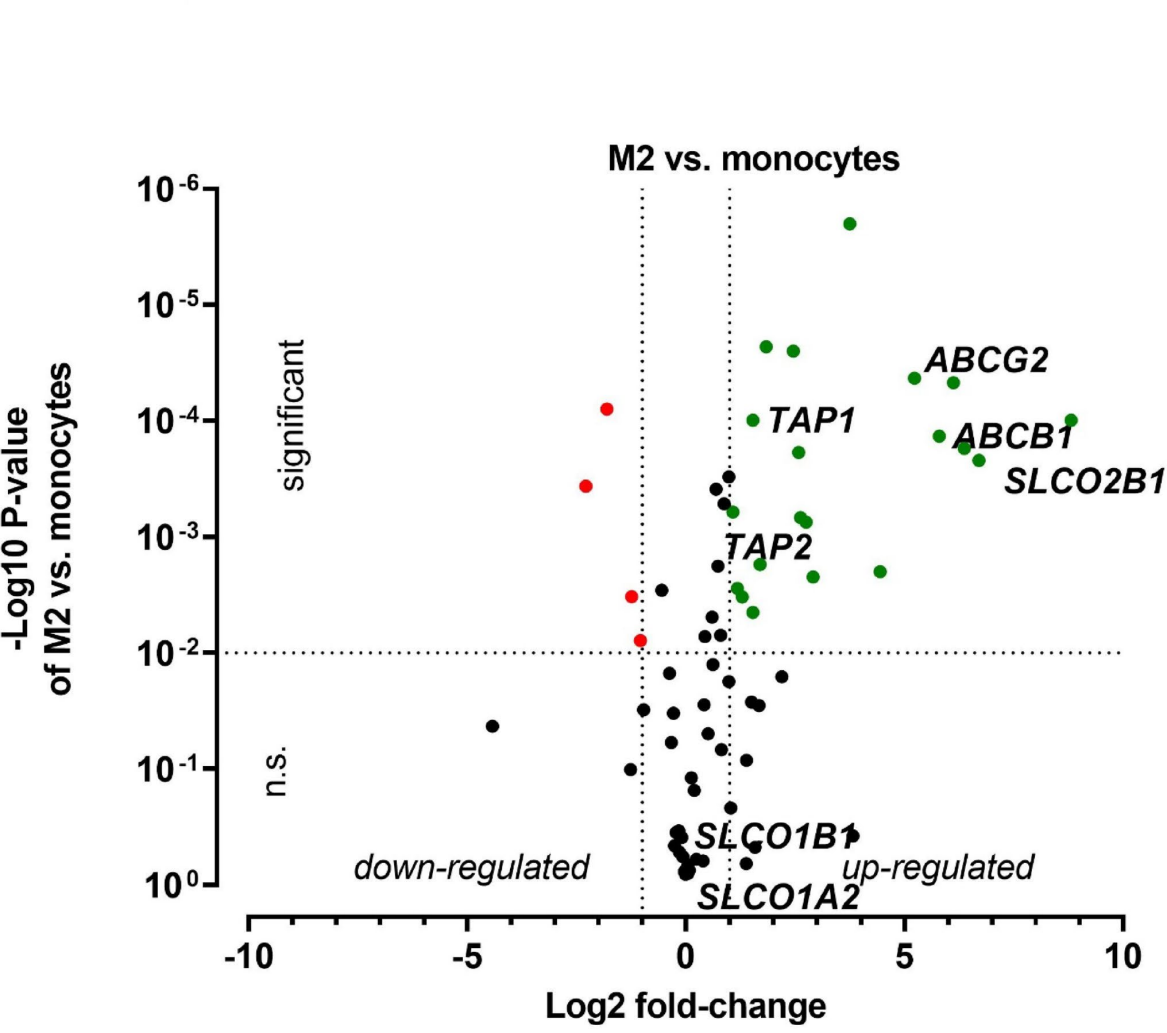
Drug transporter transcriptomics during polarization from monocytes to M2 macrophages. Expression levels were normalized to monocytes. Drug transporters were marked as green dots, if their expression was significantly (P < 0.01) increased at least two-fold compared to the control and marked red when expression was significantly decreased at least two-fold. Gene names of select drug transporters are highlighted. Data are shown as mean± S.D. of biological triplicates. Statistical significance was determined by parametric, two-sided t-test

 To investigate the transportome transcriptomics of differentiated and polarized macrophages, mRNA expression of 84 drug transporter genes was analyzed and compared to monocytes used as controls. Following the Food and Drug Administration (FDA) guidelines, only statistically significant alterations beyond two-fold were considered relevant. These genes were highlighted in the figures by green coloring (upregulation) or red coloring (downregulation) (Figs. 2, 3 and 4).

In detail, monocyte-to macrophage differentiation upregulated 18 drug transporters (Fig. 2). The most prominent alterations included *ABCB1* (56-fold), *SLCO2B1* (488-fold), *TAP1* (3-fold), and *TAP2* (2-fold). *TAP 1* and *2* (*t*ransporter associated with *a*ntigene *p*resentation 1 and 2) are needed for processing antigens for their presentation on the surface of macrophages (Lankat-Buttgereit et al. 2002). In contrast, *SLC38A5* (3-fold) and *SLC19A1* (4-fold) were the only genes downregulated in M0 macrophages.

M1 polarization enhanced 48 drug transporter genes (Fig. 3), including a 166-fold upregulation of *ABCB1*. Moreover, *TAP1* (30-fold), *SLCO1B1* (3-fold) and *SLCO2B1* (64-fold) were significantly enhanced as well. A total of seven drug transporters were downregulated, including *SLC19A1* (15-fold) and *AQP1* (7-fold).

In contrast, polarization to the M2 phenotype upregulated 20 transporter genes, most prominently reflected by *ABCG2* (37-fold), *SLCO2B1* (448-fold), *SLC7A8* (104-fold). In comparison to M1 polarization, only four genes were downregulated: *ABCA2* (2-fold), *SLC19A1* (5-fold), *SLC38A5* (3-fold) and *AQP1* (2-fold).

All data regarding fold changes and P values can be found in the supplemental material (Table S2, S3, and S4).

### M2 macrophages exhibit enhanced P-gp efflux activity

**Fig. 5:**
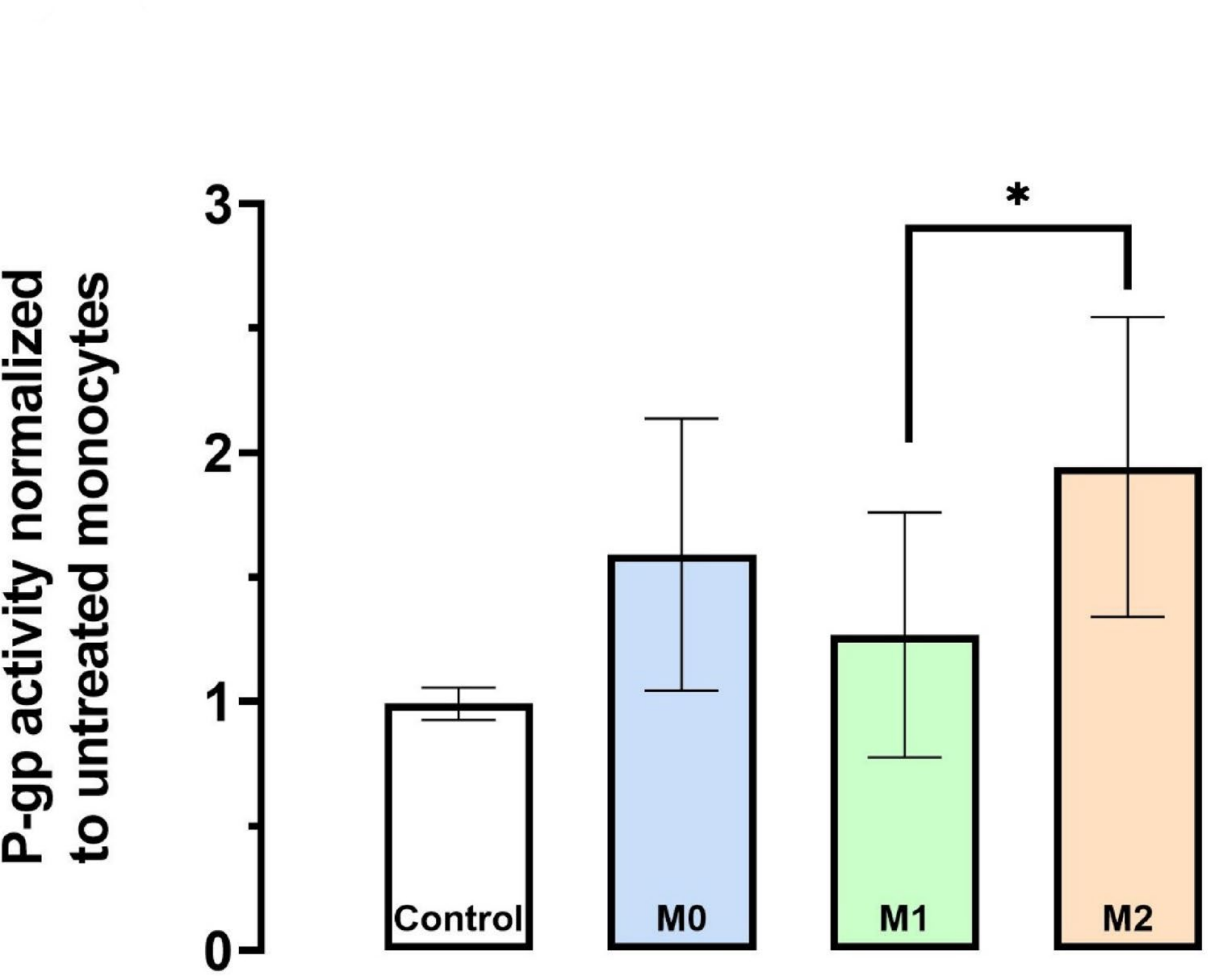
P-gp efflux activity of macrophage subtypes compared to monocytes. Cells were loaded with the fluorescent P-gp substrate rhodamine 123 and then either treated with 1 µM zosuquidar (a potent P-gp inhibitor) or left untreated. The fluorescence ratio of P-gp-inhibited to non-inhibited cells was normalized to the ratio obtained from monocytes (set to 1). Data are shown as mean ± S.D. of eight biological replicates. Statistical significance was evaluated by ANOVA with Tukey post-hoc test

 Given the considerable mRNA increase of *ABCB1* during differentiation and polarization, the corresponding efflux activity of P-gp was evaluated. For that reason, the efflux of the fluorescent P-gp surrogate substrate rhodamine 123 was recorded in all cell types analyzed and compared to monocytes (set to 1): M2 macrophages exhibited the highest P-gp efflux activity (1.9-fold higher than in monocytes), followed by differentiated M0 macrophages (1.6-fold higher than in monocytes), and M1 cells (1.3-fold higher than in monocytes) (Fig. 5). Importantly, P-gp activity in M2 cells was 1.55-fold higher than in M1 macrophages (*P* < 0.05).

### Rifampicin uptake kinetics in polarized macrophages

**Fig. 6:**
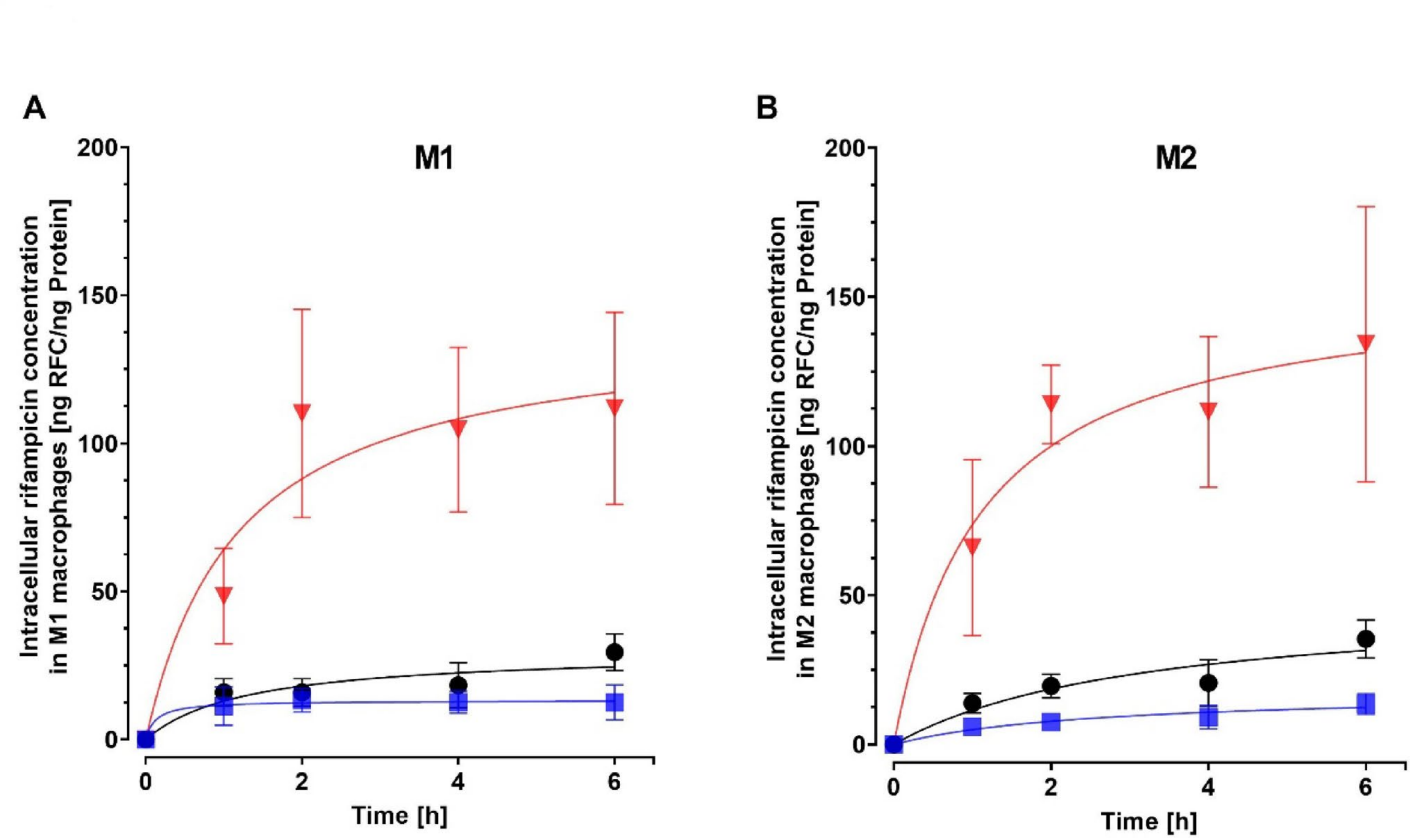
Rifampicin (RFC) uptake into M1 and M2 macrophages. Cells were exposed to 0.05 µM (blue), 0.1 µM (black), or 0.5 µM (red) rifampicin for 1 h, 2 h, 4 h, or 6 h, and resulting intracellular rifampicin concentrations (ng rifampicin/ng protein) were recorded. Data are shown as mean ± S.D. of three biological replicates. Curves were fitted through zero using hyperbola function

**Fig. 7:**
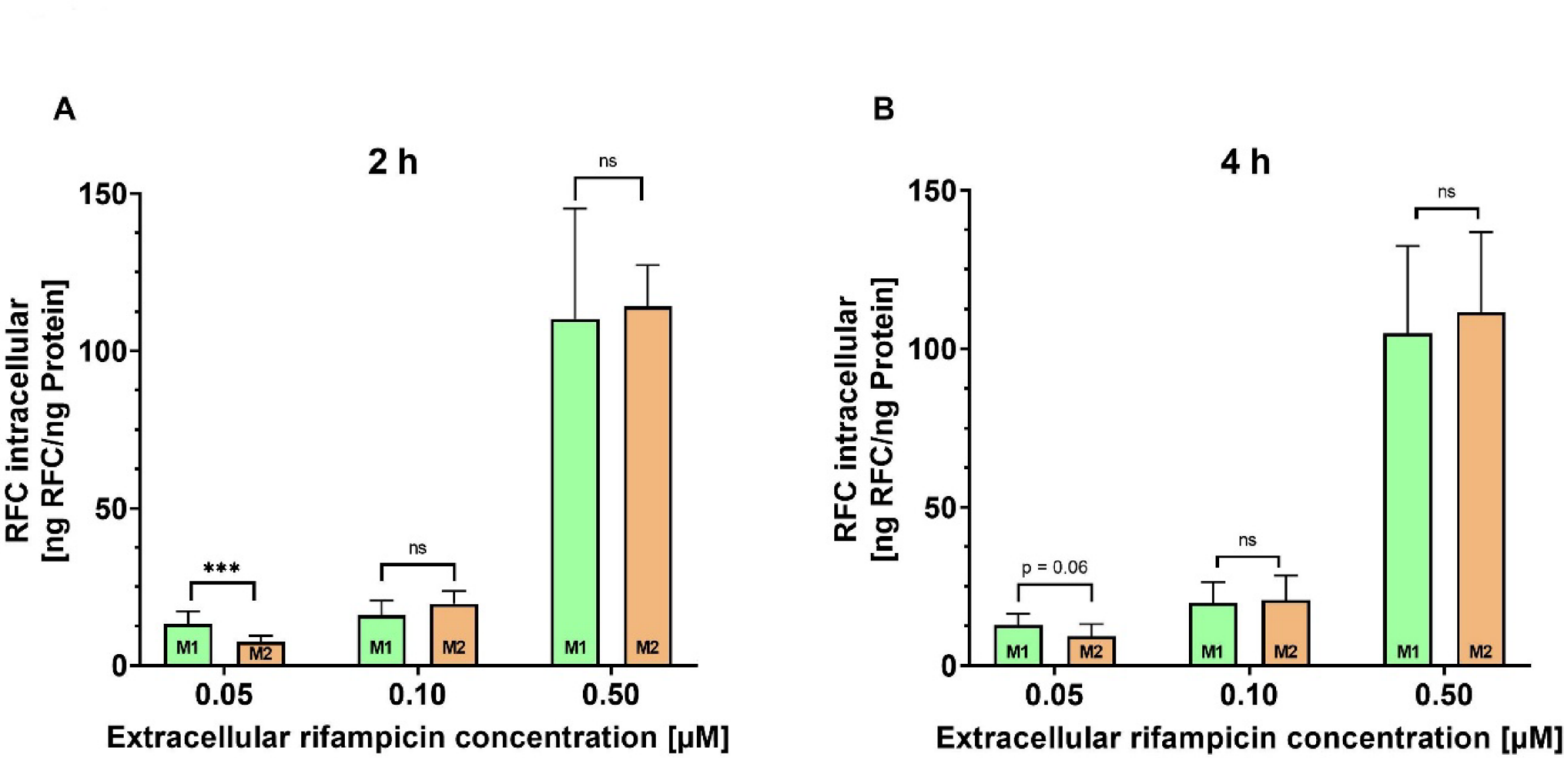
Comparison of rifampicin uptake between M1 and M2 macrophages**. **Intracellular rifampicin concentration after 2 h (**A**) and 4 h (**B**) treatment with 0.5 µM, 0.1 µM, or 0.05 µM extracellular rifampicin. Data are shown as mean ± S.D. of three biological replicates. For statistical analysis, the Mann-Whitney test was used for each extracellular rifampicin concentration

 For an objective comparison of cellular drug accumulations, steady-state conditions are required. In consequence, M1 and M2 cells were treated with different rifampicin concentrations for four different time periods and the resulting intracellular rifampicin concentrations were quantified. Rifampicin uptake was concentration-dependent for both cell types. However, while intracellular rifampicin concentrations strongly increased within the first two hours of exposure (e.g. for 0.5 µM treatment: M1, 48.5 ± 16 to 110 ± 35 ng rifampicin/ng protein; M2, 66 ± 30 to 114 ± 13 ng rifampicin/ng protein), no changes occurred thereafter (Fig. 6), suggesting that steady-state conditions are reached after 2 h at the latest. Accordingly, detailed concentration comparisons were performed for the 2 h and 4 h exposure times, respectively. When treated for 2 h with 0.05 µM rifampicin, M2 cells (7.5 ± 1.9 ng rifampicin/ng protein) had significantly lower intracellular rifampicin concentrations than M1 cells (13.3 ± 3.9 ng rifampicin/ng protein; *P* < 0.001). After 4 h, this difference remained borderline significant: While M1 macrophages accumulated 12.6 ± 3.7 ng rifampicin/ng protein, M2 cells only exhibited 9.2 ± 3.9 ng rifampicin/ng protein (*P* = 0.06). In contrast, when cells were exposed to higher extracellular concentrations (0.1 µM or 0.5 µM rifampicin), resulting intracellular concentrations after 2–4 h did not differ between M1 and M2 cells (Fig. 7).

## Discussion

The aim of this study was to investigate the impact of polarization of THP-1 monocytes into M1 and M2 macrophages on drug transporter transcriptomics and whether the resulting higher P-gp efflux activity in M2 macrophages could impair rifampicin uptake compared to M1 macrophages. This seems relevant because M2 macrophages are a beneficial niche for Mtb bacteria, fostering their proliferation and immune escape (Lastrucci et al. 2015). Moreover, M2-dominant tuberculosis has been associated with poor outcome and antibiotic treatment resistance, calling for the evaluation of drug penetration into M2 cells and the underlying cellular mechanisms modulating drug uptake. In consequence, this experimental study pursued the following approach.

First, we needed to confirm the successful generation of the correct polarization phenotypes by evaluating the well-established polarization markers TNFα and CCL22 (Shiratori et al. 2017) As expected, pro-inflammatory *TNFα* was significantly increased in M1 macrophages and anti-inflammatory *CCL22* was higher expressed in M2 macrophages. Secondly, after confirming the successful differentiation and polarization, the very same mRNA was used to test for 84 different drug transporters in each macrophage phenotype (M0, M1 and M2). The data clearly demonstrated that monocyte-to-macrophage differentiation already induces the mRNA expression of a broad set of drug transporters. This trend continues during polarization to M1 and to M2 macrophages. Most profoundly, *ABCB1* was 166-fold enhanced in M1 cells and 55-fold increased in M2 macrophages (compared to monocytes). Third, because P-gp is known to modulate the uptake of its substrates into immune cells (Hasanuzzaman et al. 2019), its efflux activity level was recorded using a fluorescent substrate and flow cytometry. In line with the mRNA expression levels, both M1 and M2 cells exhibited higher P-gp activities than monocytes. The uptake of rifampicin into these cells is thus expected to be lower than in non-P-gp-expressing cells (e.g. monocytes). However, because enhanced P-gp activity can be blunted by concurrent enhancement of drug influx activity (e.g. mediated by influx transporters such as *SLCO2B1* or *SLCO1B1*), the actual net uptake of rifampicin into target cells was quantified by UPLC-MS/MS. Forth and eventually, this data demonstrated that M2 cells take up less rifampicin when cells are extracellularly exposed to 0.05 µM. But is 0.05 µM a clinically relevant exposure? During chronic treatment with standard doses of rifampicin (e.g. 10 mg/kg), through levels (C_min_) of total rifampicin under steady-state conditions in the plasma is about 0.006 µM (0.005 µg/mL) (Chirehwa et al. 2015). The distribution of rifampicin into the epithelial lining fluid of the lung is about 30% (Clewe et al. 2015; Ziglam et al. 2002), leading to a theoretical steady-state rifampicin C_min_ at the site of action of approximately 0.002 µM. Together, this indicates that the experimental rifampicin concentration used in our uptake experiments (0.05 µM) is likely of clinical relevance, albeit rather being at the higher end of expected in vivo rifampicin concentrations. In contrast, treatment of macrophages with very high, clinically non-relevant rifampicin concentrations masked the uptake differences between M2 cells and M1 cells. This indicates that active efflux transporters (e.g. P-gp) can in fact prevent penetration of low concentrations (e.g. 0.05 µM) but hardly counteract high extracellular concentrations of the lipophilic compound rifampicin, which is known for its pronounced cellular accumulation (Nilles et al. 2023, 2024; Tanner et al. 2021). During long-term treatment with rifampicin, P-gp expression and activity in macrophages could theoretically increase further on, given rifampicin’s well-known transcriptional effects on *ABCB1*, at least in intestinal or hepatic tissues. However, data on the *ABCB1*/P-gp-regulating effects of rifampicin in immune cells are indefinite or contradicting, likely depending on the route of rifampicin exposure (in vivo vs. *ex vivo;* Asghar et al. 2002; Owen et al. 2006; Manceau et al. 2010). For differentiated M0 macrophages, Hasanuzzaman and co-workers demonstrated *ABCB1*/P-gp induction after substantial rifampicin treatment (100 µM for 48 h) and explained it by rifampicin’s action on the nuclear transcription factor called pregnane X receptor (PXR) (Hasanuzzaman et al. 2019). For polarized macrophages (M1 or M2), there is no such data at the moment.

Besides transcriptional effects (either mediated by differentiation, polarization, or rifampicin treatment), actual changes of anti-tuberculosis drug uptake are likely of higher clinical relevance. So far, only differentiated M0 macrophages have been characterized for the uptake of prothionamide (Hasanuzzaman et al. 2019), isoniazid, ethambutol, or rifampicin (Hartkoorn et al. 2007). Again, data on the uptake of anti-tuberculosis drugs into polarized M1 or M2 macrophages is lacking. Accordingly, the presented study was the first to investigate rifampicin uptake into this very specific cell type, that is relevant for clinical efficacy.

This study has its limitations. First, a commercial monocyte cell line was used for all experiments. While THP-1 is a well-known and established model for monocytes or derived macrophages (Chanput et al. 2014), generalization to in vivo conditions is likely hard to do. Second, the used M1/M2 model is a simplification of the actual biological spectrum of polarized macrophages (Mosser et al., 2008). Moreover, there certainly is a cross-talk between polarized macrophages and the Mtb pathogen, that comprises PXR-mediated transcriptional effects of Mtb constituents (e.g. fatty acids) on drug transporters (Bhagyaraj et al. 2016; Hudson et al. 2019; Refai et al. 2018). However, this cross-talk was not experimentally captured in our work. Third, only P-gp was evaluated on a functional level. Thus, possible influences of other drug transporters remain unclear.

Despite these limitations, the work presented here also has clear scientific or methodological strengths. First, this is the first data set comprising a high number of different drug transporters in THP-1-derived polarized macrophages. The pool of tested transporters in previous studies was lower (45 vs. 84) and compared granulo-macrophagic colony-stimulating factor-generated macrophages to parental blood monocytes (Moreau et al. 2011) Second, this evaluation of drug transporter transcriptional changes during all important steps of monocyte differentiation and polarization is not only valuable for tuberculosis antibiosis, but potentially helpful in the framework of tumor-associated macrophages, being important factors of tumor disease outcome and a potential target for antineoplastic drugs (Bai et al. 2024; Li et al. 2024; Guan et al. 2025). Consequently, the data presented here could promote research in anti-cancer drug treatments of macrophage-enriched tumor environments, given the importance of P-gp for anti-cancer drug transport out of tumor cells and immune cells (Robinson 2020). Third, we measured P-gp efflux activity in THP-1-derived polarized macrophages using rhodamine 123 (P-gp substrate) and zosuquidar (P-gp inhibitor). Previous investigations either used other cell models (U937 cell line, Cory et al. 2016; RAW264.7 cells, Zha et al. 2013; THP-1-derived M0 macrophages treated with rifampicin, Hasanuzzaman et al. 2019) or other P-gp surrogate substrates such as Hoechst 33,342 (Cory et al. 2016), which is considered rather a substrate for BCRP (Kim et al. 2002). Also, previous investigations used diverse other P-gp inhibitors (e.g. elacridar, Cory et al. 2016; cyclosporin A, Hasanuzzaman et al. 2019; protease inhibitors or verapamil, Zha et al. 2013), while we used the established P-gp inhibitor zosuquidar (Dantzig et al. 1996), that is quite P-gp-specific at 1 µM (Bajraktari-Sylejmani et al. 2025). Accordingly, our sound data on P-gp in polarized macrophages can certainly be extrapolated to other anti-tuberculosis drugs being transported by P-gp such as bedaquiline (Kotwal et al. 2020), moxifloxacin (Brillault et al. 2009), and prothionamide (Hasanuzzaman et al. 2019). Forth and finally, actual cellular rifampicin kinetics in M1 and M2 cells was recorded, including the evaluation of intracellular steady-state concentrations, being proven to be reached after 2 h at the latest. In consequence, researchers should treat their monocyte-derived macrophages for at least 2 h to ensure saturated intracellular drug levels.

## Conclusion

This is the first study to investigate the transcriptomic variability of numerous drug transporters, P-gp activity, and rifampicin uptake kinetics in polarized M1 and M2 macrophages, derived from THP-1 monocytes. The obtained data suggests that poor rifampicin efficacy in M2-dominant tuberculosis is at least in part related to higher efflux transport resulting in lowered rifampicin intracellular bioavailability, given the polarization-dependent alterations of drug transporter expressions and functions, including an enhanced activity of P-gp.

## Supplementary Information

Below is the link to the electronic supplementary material.

Supplementary Material 1
