# Supplementary material for: THP-1-derived polarized macrophages differ by drug transporter transcriptomics, P-glycoprotein activity and rifampicin uptake kinetics
Source: Arch Toxicol. 2026 Jan 9;100(4):1377–89. doi: 10.1007/s00204-025-04270-2 (PMC13043547; doi:10.1007/s00204-025-04270-2)
Supplement: Supplementary file 1 — Supplementary Material 1 [file 204_2025_4270_MOESM1_ESM.docx]

**Supplementary Material**

**Table S1: Primer sequences of reference genes and polarization markers used for qPCR.**

| **Genes** | **Forward Primer** | **Reverse Primer** | **Reference** |
| --- | --- | --- | --- |
| *GU* | 5´-TTCACCAGGATCCACCTCTG | 5´-TGTTCAAACTTCTGCTCCTG | Weiss et al., 2008 |
| *RPL13* | 5´-GCTCATGAGGCTACGGAAAC | 5´-TATTGGGCTCAGACCAGGAG | Weiss et al., 2008 |
| *TNFα* | 5′-TGG AGC TGG CCG AGG AG | 5′-AGC AGG CAG AAG AGC GTG G | Shiratori et al., 2017 |
| *CCL22* | 5′-ATT ACG TCC GTT ACC GTC TG | 5′-TAG GCT CTT CAT TGG CTC AG | Shiratori et al., 2017 |

**Table S2: Fold-change and P values of drug transporter genes** **of differentiated M0 macrophages normalized** **to monocytes.**

| **Gene** | **Log2 Fold change** | **P value** |
| --- | --- | --- |
| *ABCA1* | 1.53106949272595 | 0.012472 |
| *ABCA12* | 0.985500430304885 | 0.355252 |
| *ABCA13* | 3.05137210172103 | 0.000411 |
| *ABCA3* | 1.16992500144231 | 0.015831 |
| *ABCA4* | 6.50874555424448 | 0.001901 |
| *ABCA5* | 1.96347412397489 | 0.712736 |
| *ABCA9* | 0.863938450423972 | 0.278827 |
| *ABCB1* | 5.80864246791807 | 0.000035 |
| *ABCB4* | 1.87184364850932 | 0.000166 |
| *ABCB5* | 1.89917563048051 | 0.031011 |
| *ABCB6* | 0.111031312388744 | 0.678273 |
| *ABCC1* | 1.38956681176273 | 0.0144 |
| *ABCC10* | 1.10433665981474 | 0.134031 |
| *ABCC12* | 1.31034012061215 | 0.211615 |
| *ABCC2* | 0.356143810225275 | 0.469535 |
| *ABCC3* | 6.59200846130731 | 0.002426 |
| *ABCC5* | 1.4541758931858 | 0.025856 |
| *ABCD1* | 0.16349873228288 | 0.493177 |
| *ABCD3* | 3.82578562746479 | 0.373483 |
| *ABCD4* | 1.16992500144231 | 0.012513 |
| *ABCF1* | 0.310340120612151 | 0.16416 |
| *ABCG2* | 5.37851162325373 | 0.00134 |
| *ABCG8* | 1.77820857639809 | 0.131388 |
| *AQP7* | 1.31034012061215 | 0.211615 |
| *AQP9* | 2.75274859140713 | 0.040593 |
| *ATP6V0C* | 0.432959407276106 | 0.102132 |
| *ATP7A* | 1.81966818349646 | 0.000464 |
| *MVP* | 1.18903382439002 | 0.004107 |
| *SLC10A1* | 1.83995958748953 | 0.359828 |
| *SLC10A2* | 1.31034012061215 | 0.211615 |
| *SLC15A1* | 1.40599235967584 | 0.240051 |
| *SLC16A1* | 0.189033824390017 | 0.403356 |
| *SLC16A2* | 2.63923216324928 | 0.000059 |
| *SLC16A3* | 1.13093086982645 | 0.196014 |
| *SLC19A2* | 0.516015147003665 | 0.008234 |
| *SLC19A3* | 1.31034012061215 | 0.211615 |
| *SLC22A1* | 1.31034012061215 | 0.211615 |
| *SLC22A2* | 1.31034012061215 | 0.211615 |
| *SLC22A3* | 1.31034012061215 | 0.211615 |
| *SLC22A6* | 1.37295209791183 | 0.230729 |
| *SLC22A7* | 1.31034012061215 | 0.211615 |
| *SLC22A8* | 1.31034012061215 | 0.211615 |
| *SLC22A9* | 1.35614381022528 | 0.219806 |
| *SLC28A1* | 1.37295209791183 | 0.191099 |
| *SLC28A2* | 1.31034012061215 | 0.211615 |
| *SLC28A3* | 4.58856473740135 | 0.001017 |
| *SLC29A1* | 0.37851162325373 | 0.088587 |
| *SLC29A2* | 0.815575428862573 | 0.386313 |
| *SLC2A1* | 0.948600847493356 | 0.04805 |
| *SLC2A2* | 1.31034012061215 | 0.211615 |
| *SLC2A3* | 2.90496571868403 | 0.004396 |
| *SLC31A1* | 2.55581615506164 | 0.000004 |
| *SLC38A2* | 0.189033824390017 | 0.137774 |
| *SLC3A1* | 1.55581615506164 | 0.1282 |
| *SLC3A2* | 0.895302621333307 | 0.021659 |
| *SLC5A1* | 1.31034012061215 | 0.211615 |
| *SLC5A4* | 1.58976348698498 | 0.111093 |
| *SLC7A5* | 0.250961573533219 | 0.289209 |
| *SLC7A6* | 0.613531652917927 | 0.040649 |
| *SLC7A7* | 3.76871365703049 | 0.000107 |
| *SLC7A8* | 6.59872249967662 | 0.000227 |
| *SLC7A9* | 1.31034012061215 | 0.211615 |
| *SLCO1A2* | 1.31034012061215 | 0.211615 |
| *SLCO1B1* | 1.31034012061215 | 0.211615 |
| *SLCO1B3* | 1.31034012061215 | 0.211615 |
| *SLCO2A1* | 1.31034012061215 | 0.211615 |
| *SLCO2B1* | 8.92534673617656 | 0.001911 |
| *SLCO3A1* | 0.75702324650746 | 0.022818 |
| *SLCO4A1* | 2.58976348698498 | 0.000279 |
| *TAP1* | 1.72246602447109 | 0.002168 |
| *TAP2* | 1.08406426478847 | 0.003134 |
| *B2M* | 1.36737106564853 | 0.000214 |
| *ABCA2* | -0.659924558 | 0.175757 |
| *ABCB11* | -3.09085343 | 0.056841 |
| *ABCC11* | -0.042644337 | 0.822898 |
| *ABCC4* | -0.333423734 | 0.084747 |
| *AQP1* | -1.117695043 | 0.017745 |
| *ATP7B* | -0.298658316 | 0.213078 |
| *SLC15A2* | -0.526068812 | 0.352122 |
| *SLC19A1* | -1.922197848 | 0.00151 |
| *SLC25A13* | -0.084064265 | 0.603152 |
| *SLC38A5* | -1.555816155 | 0.000209 |
| *SLC7A11* | -0.084064265 | 0.744301 |
| *VDAC1* | -0.189033824 | 0.109849 |
| *VDAC2* | -0.014355293 | 0.867719 |

**Table S3: Fold-change and P values of drug transporter genes** **of M1 macrophages normalized** **to monocytes.**

| **Gene** | **Log2 Fold change** | **P value** |
| --- | --- | --- |
| *ABCA1* | 2.92789645372882 | 0.002376 |
| *ABCA12* | 0.321928094887362 | 0.680136 |
| *ABCA13* | 5.06522762277562 | 0.000073 |
| *ABCA3* | 0.310340120612151 | 0.022774 |
| *ABCA4* | 7.78868571061354 | 0.000478 |
| *ABCA5* | 1.57046293102604 | 0.476597 |
| *ABCA9* | 2.65306001710456 | 0.000008 |
| *ABCB1* | 7.38870616922638 | 0.000015 |
| *ABCB4* | 1.20163386116965 | 0.003443 |
| *ABCB5* | 1.82374936030827 | 0.001046 |
| *ABCB6* | 0.36737106564853 | 0.113532 |
| *ABCC1* | 1.60880924267552 | 0.000114 |
| *ABCC10* | 1.54101915313356 | 0.000021 |
| *ABCC11* | 0.485426827170242 | 0.436667 |
| *ABCC12* | 1.75702324650746 | 0.037853 |
| *ABCC3* | 7.19199684666977 | 0.000003 |
| *ABCC5* | 0.641546029087524 | 0.024557 |
| *ABCD1* | 1.62293035092018 | 0.000485 |
| *ABCD3* | 3.0686708106651 | 0.838037 |
| *ABCD4* | 1 | 0.000068 |
| *ABCF1* | 0.748461233004036 | 0.007832 |
| *ABCG2* | 3.65191274464578 | 0.000008 |
| *ABCG8* | 2.16349873228288 | 0.038912 |
| *AQP7* | 1.93734439215023 | 0.000684 |
| *AQP9* | 4.03562390973072 | 0.000741 |
| *ATP6V0C* | 1.61353165291793 | 0.000145 |
| *ATP7A* | 1.92219784839637 | 0.001544 |
| *MVP* | 3.70154905694307 | 0.000004 |
| *SLC10A1* | 3.00539998774259 | 0.03487 |
| *SLC10A2* | 1.57531233068744 | 0.008144 |
| *SLC15A1* | 1.7311832415722 | 0.004644 |
| *SLC16A2* | 1.94110631094643 | 0.000017 |
| *SLC16A3* | 2.08746284125034 | 0.00304 |
| *SLC19A2* | 1.75702324650746 | 0.000003 |
| *SLC19A3* | 1.57531233068744 | 0.008144 |
| *SLC22A1* | 2.88166461932035 | 0.029065 |
| *SLC22A2* | 1.88752527074159 | 0.00361 |
| *SLC22A3* | 1.57531233068744 | 0.008144 |
| *SLC22A6* | 1.90689059560852 | 0.00514 |
| *SLC22A7* | 1.83187724119167 | 0.001192 |
| *SLC22A8* | 1.57531233068744 | 0.008144 |
| *SLC22A9* | 2.84197311892718 | 0.007258 |
| *SLC28A1* | 2.32481060342048 | 0.000848 |
| *SLC28A2* | 2 | 0.001181 |
| *SLC28A3* | 6.58871463558226 | 0.000122 |
| *SLC29A2* | 0.111031312388744 | 0.806159 |
| *SLC2A1* | 0.992768430768924 | 0.007628 |
| *SLC2A2* | 1.61353165291793 | 0.004271 |
| *SLC2A3* | 3.37851162325373 | 0.000063 |
| *SLC31A1* | 3.85199883711245 | 0.000006 |
| *SLC38A2* | 0.807354922057604 | 0.00104 |
| *SLC3A1* | 1.55090066464752 | 0.121758 |
| *SLC3A2* | 1.57046293102604 | 0.000014 |
| *SLC5A1* | 1.62293035092018 | 0.011765 |
| *SLC5A4* | 2.4541758931858 | 0.023212 |
| *SLC7A11* | 3.22496636500027 | 0.00026 |
| *SLC7A6* | 0.250961573533219 | 0.065024 |
| *SLC7A7* | 3.23572705983806 | 0.000086 |
| *SLC7A8* | 5.59185890498717 | 0.000008 |
| *SLC7A9* | 1.57531233068744 | 0.008144 |
| *SLCO1A2* | 1.57531233068744 | 0.008144 |
| *SLCO1B1* | 1.57531233068744 | 0.008144 |
| *SLCO1B3* | 1.57531233068744 | 0.008144 |
| *SLCO2A1* | 1.82374936030827 | 0.058911 |
| *SLCO2B1* | 5.90857275875175 | 0.002648 |
| *SLCO3A1* | 0.310340120612151 | 0.028687 |
| *SLCO4A1* | 3.23572705983806 | 0.000001 |
| *TAP1* | 4.92884403671257 | 0.000067 |
| *TAP2* | 3.01792190799726 | 0.000173 |
|  |  |  |
|  |  |  |
| *ABCA2* | -1.63227 | 0.002875 |
| *ABCB11* | -2.82782 | 0.055273 |
| *ABCC2* | -0.08406 | 0.772117 |
| *ABCC4* | -2.02857 | 0.000034 |
| *AQP1* | -2.73769 | 0.00041 |
| *ATP7B* | -0.36737 | 0.274673 |
| *SLC15A2* | -2.73552 | 0.020887 |
| *SLC16A1* | -1.02857 | 0.000192 |
| *SLC19A1* | -3.86096 | 0.000188 |
| *SLC25A13* | -0.27501 | 0.088831 |
| *SLC29A1* | -0.76553 | 0.00023 |
| *SLC38A5* | -2.27798 | 0.000048 |
| *SLC7A5* | -1.01436 | 0.000315 |
| *VDAC1* | -0.50589 | 0.00473 |
| *VDAC2* | -0.04264 | 0.659437 |

**Table S4: Fold-change and P values of drug transporter genes** **of M2 macrophages normalized** **to monocytes.**

| **Gene** | **Log2 Fold change** | **P value** |
| --- | --- | --- |
| *ABCA1* | 1.52606881166759 | 0.004451 |
| *ABCA12* | 0.389566811762726 | 0.61947 |
| *ABCA13* | 2.90496571868403 | 0.002204 |
| *ABCA3* | 0.978195629681652 | 0.000305 |
| *ABCA4* | 6.11790278909403 | 0.000047 |
| *ABCA5* | 1.57531233068744 | 0.474447 |
| *ABCA9* | 0.070389327891398 | 0.745325 |
| *ABCB1* | 5.79467578798686 | 0.000136 |
| *ABCB4* | 1.83187724119167 | 0.000023 |
| *ABCB5* | 1.49569516262407 | 0.026427 |
| *ABCC1* | 1.17632277264046 | 0.002771 |
| *ABCC10* | 0.505890929729957 | 0.049608 |
| *ABCC3* | 6.36806987653208 | 0.000173 |
| *ABCC5* | 1.0703893278914 | 0.000613 |
| *ABCD3* | 3.82171021503467 | 0.376531 |
| *ABCD4* | 0.871843648509318 | 0.000519 |
| *ABCF1* | 0.0426443374084937 | 0.793108 |
| *ABCG2* | 5.22458056608344 | 0.000043 |
| *ABCG8* | 0.978195629681652 | 0.017599 |
| *AQP9* | 2.19219416528335 | 0.016058 |
| *ATP6V0C* | 0.613531652917927 | 0.012575 |
| *ATP7A* | 1.6959938131099 | 0.001725 |
| *MVP* | 1.28688114778816 | 0.003278 |
| *SLC10A1* | 1.37851162325373 | 0.653275 |
| *SLC16A1* | 0.189033824390017 | 0.152669 |
| *SLC16A2* | 2.74846123300404 | 0.000748 |
| *SLC16A3* | 0.807354922057604 | 0.068064 |
| *SLC19A2* | 0.432959407276106 | 0.007224 |
| *SLC22A9* | 1.38404980679516 | 0.084249 |
| *SLC28A3* | 4.44161626940656 | 0.001989 |
| *SLC29A1* | 0.124328135002202 | 0.119106 |
| *SLC2A1* | 0.790772037862 | 0.007016 |
| *SLC2A2* | 0.238786859587117 | 0.601642 |
| *SLC2A3* | 2.61823865559546 | 0.000682 |
| *SLC31A1* | 2.57773093149008 | 0.000187 |
| *SLC38A2* | 0.0426443374084937 | 0.672992 |
| *SLC3A1* | 1.02147972741045 | 0.216177 |
| *SLC3A2* | 0.687060688339893 | 0.000388 |
| *SLC5A4* | 1.66902676550963 | 0.028292 |
| *SLC7A6* | 0.411426245726465 | 0.027828 |
| *SLC7A7* | 3.74416109557041 | 0.000002 |
| *SLC7A8* | 6.70127180220197 | 0.000219 |
| *SLCO2B1* | 8.81134257974037 | 0.000099 |
| *SLCO3A1* | 0.594548549550354 | 0.004907 |
| *SLCO4A1* | 2.45680614923047 | 0.000025 |
| *TAP1* | 1.52606881166759 | 0.000099 |
| *TAP2* | 0.7311832415722 | 0.001778 |
| *B2M* | 1.4541758931858 | 0 |
| *ABCA2* | -1.042644337 | 0.007792 |
| *ABCB11* | -4.428946345 | 0.04275 |
| *ABCB6* | -0.176322773 | 0.362572 |
| *ABCC11* | -1.269033146 | 0.101408 |
| *ABCC12* | -0.028569152 | 0.767837 |
| *ABCC2* | -0.263034406 | 0.457765 |
| *ABCC4* | -0.555816155 | 0.002879 |
| *ABCD1* | -0.070389328 | 0.570812 |
| *AQP1* | -1.244887059 | 0.00327 |
| *AQP7* | -0.028569152 | 0.767837 |
| *ATP7B* | -0.333423734 | 0.059028 |
| *SLC10A2* | -0.028569152 | 0.767837 |
| *SLC15A1* | -0.028569152 | 0.767837 |
| *SLC15A2* | -0.970853654 | 0.030907 |
| *SLC19A1* | -2.292781749 | 0.000366 |
| *SLC19A3* | -0.028569152 | 0.767837 |
| *SLC22A1* | -0.028569152 | 0.767837 |
| *SLC22A2* | -0.028569152 | 0.767837 |
| *SLC22A3* | -0.028569152 | 0.767837 |
| *SLC22A6* | -0.028569152 | 0.767837 |
| *SLC22A7* | -0.028569152 | 0.767837 |
| *SLC22A8* | -0.028569152 | 0.767837 |
| *SLC25A13* | -0.378511623 | 0.014954 |
| *SLC28A1* | -0.014355293 | 0.808548 |
| *SLC28A2* | -0.028569152 | 0.767837 |
| *SLC29A2* | -0.150559677 | 0.5214 |
| *SLC38A5* | -1.807354922 | 0.000079 |
| *SLC5A1* | -0.028569152 | 0.767837 |
| *SLC7A11* | -0.22650853 | 0.351895 |
| *SLC7A5* | -0.163498732 | 0.340365 |
| *SLC7A9* | -0.028569152 | 0.767837 |
| *SLCO1A2* | -0.028569152 | 0.767837 |
| *SLCO1B1* | -0.028569152 | 0.767837 |
| *SLCO1B3* | -0.028569152 | 0.767837 |
| *SLCO2A1* | -0.028569152 | 0.767837 |
| *VDAC1* | -0.286881148 | 0.032997 |
| *VDAC2* | -0.097610797 | 0.390435 |
